# Complete Genome Sequence of a *Luteibacter* Strain Isolated from a Carnivorous Pitcher Plant (Sarracenia minor)

**DOI:** 10.1128/mra.00697-22

**Published:** 2022-11-03

**Authors:** Timothy Yaroshuk, Nikolas M. Stasulli

**Affiliations:** a Department of Forensic Science, University of New Haven, West Haven, Connecticut, USA; b Department of Biology and Environmental Science, University of New Haven, West Haven, Connecticut, USA; University of Arizona

## Abstract

A bacterial isolate of Luteibacter anthropi, designated SM7.4, was isolated from decaying insect detritus inside a carnivorous pitcher plant (Sarracenia minor). Here, we report a complete genome sequence for Luteibacter anthropi strain SM7.4, assembled by combining the Oxford Nanopore MinION flow cell and paired-end Illumina sequencing approaches.

## ANNOUNCEMENT

*Luteibacter* species are Gram-negative, yellow-pigmented bacteria commonly associated with the plant rhizosphere (1–3). The available genomic information for the type strain of Luteibacter anthropi is a contig level draft assembly (GenBank accession number GCA_011759365.1) that may not provide the necessary information for studying this bacteria via mutagenesis (4).

Strain SM7.4 was isolated on 17 June 2016 from pitcher plant (Sarracenia minor) insect detritus from the North Carolina Botanical Gardens (Chapel Hill, NC, USA; 35.8993°N, 79.0334°W). After incubation at 30°C, the sample was isolated and subcultured on International *Streptomyces* Project 2 (ISP-2) (5) agar plates, made to half-strength (0.5×). Isolate SM7.4 was maintained in 20% glycerol at −80°C. For genomic extractions, a pure isolate was inoculated into 50 mL 0.5× ISP-2 broth and grown overnight at 30°C, with shaking at 160 rpm. DNA was isolated using the Monarch genomic DNA purification kit (New England Biolabs) and quantified via the Invitrogen Qubit double-stranded DNA (dsDNA) high-sensitivity (HS) assay kit, all following the manufacturers’ protocols.

For Oxford Nanopore Technologies (ONT) sequencing, library preparation was completed using 200 ng of nonsheared high-molecular-weight DNA with the SQK-RAD004 rapid sequencing kit, according to the manufacturer’s instructions, on an R9.4.1 flow cell. MinKNOW v20.06.4 software was used to control the sequencing operations, and Guppy v4.0.9 was used with high accuracy for base calling. For quality control, bases with a score of ≥Q7 were sorted into a “fastq_pass” folder and used for subsequent analyses. A total of ~79,570 reads were generated, with 928.64 Mb passing the initial quality filtering (coverage, ~186×; estimated genome size, ~5 Mb). The resulting *N*_50_ value was 22.63 kb.

The same DNA sample was sequenced with an Illumina NovaSeq instrument through Novogene. For library preparation, DNA was randomly sheared to ~350 bp. Following the manufacturer’s protocol, the NEBNext DNA library prep kit was used for end repairing, dA-tailing, and NEBNext adapter ligation. Enrichment via PCR was completed on P5- and P7-indexed oligonucleotides. Paired-end (PE) sequencing generated a read length of 150 bp at each end. Base calling was completed using Casava. Overall, 5,625,594 raw reads were generated, resulting in 1.7 Gb of data (coverage, ~340×). The raw data were filtered by Novogene in-house. Briefly, paired-end reads with >10% uncertain nucleotides, adapter contamination, or >50% of nucleotides with Q ≤ 5 were discarded. The cleaned reads were used for downstream analysis.

Hybrid assembly was completed using Trycycler v0.5.0 with polishing (6). Adapter trimming was conducted using Porechop v0.2.4 (7) with default parameters. Steps 1 to 7 were performed of the Trycycler instructions on GitHub. For step 1, the assemblers Flye v2.8.3-b1695 (8), Miniasm v0.3-r179/Minipolish v0.1.2 (9), and Raven v1.5.0 (10) were used. One single cluster resulted. One contig of 12 was removed to allow for reconciliation. Medaka v1.4.1 (https://github.com/nanoporetech/medaka) was used for polishing with model r941_min_high_360. Pilon v1.24 polishing (11) was conducted 3 times. No custom scripts were used for this assembly. The final assembly was a single circular chromosome of 4,961,663 bp, which was annotated via the DFAST annotation pipeline (12). DFAST was used to determine the highest similarity to Luteibacter anthropi strain CCUG25036 and to predict a GC content of 64.2%, 4,255 protein-coding genes (CDSs), 6 rRNAs, 57 tRNAs, and 2 CRISPRs. Using ResFinder (13) with default parameters, no antibiotic resistance genes were indicated.

### Data availability.

This genome project has been deposited at GenBank under BioProject accession number PRJNA738930. The complete genome sequence described here can be found under GenBank accession number CP077072, with the genome described here being version CP077072.1. The raw sequences were deposited in the Sequence Read Archive (SRA); the raw ONT-passed fastq files are indexed under accession number SRR15460238, and the paired-end cleaned Illumina reads, as described above, are indexed under accession number SRR15460237.

## References

[1] Johansen JE, Binnerup SJ, Kroer N, Mølbak L. 2005. Luteibacter rhizovicinus gen. nov., sp. nov., a yellow-pigmented gammaproteobacterium isolated from the rhizosphere of barley (Hordeum vulgare L.). Int J Syst Evol Microbiol 55:2285–2291. doi:10.1099/ijs.0.63497-0. 16280484

[2] Akter S, Huq MA. 2018. Luteibacter pinisoli sp. nov., a casein degrading bacterium isolated from rhizospheric soil of Pinus koraiensis. Arch Microbiol 200:1017–1023. doi:10.1007/s00203-018-1515-1. 29637289

[3] Wang L, Wang G, Li S, Jiang J. 2011. Luteibacter jiangsuensis sp. nov.: a methamidophos-degrading bacterium isolated from a methamidophos-manufacturing factory. Curr Microbiol 62:289–295. doi:10.1007/s00284-010-9707-1. 20628745

[4] Kämpfer P, Lodders N, Falsen E. 2009. Luteibacter anthropi sp. nov., isolated from human blood, and reclassification of Dyella yeojuensis Kim et al. 2006 as Luteibacter yeojuensis comb. nov. Int J Syst Evol Microbiol 59:2884–2887. doi:10.1099/ijs.0.009100-0. 19628591

[5] Shirling EB, Gottlieb D. 1966. Methods for characterization of Streptomyces species 1. Int J Syst Evol Microbiol 16:313–340. doi:10.1099/00207713-16-3-313.

[6] Wick RR, Judd LM, Cerdeira LT, Hawkey J, Méric G, Vezina B, Wyres KL, Holt KE. 2021. Trycycler: consensus long-read assemblies for bacterial genomes. Genome Biol 22:266. doi:10.1186/s13059-021-02483-z. 34521459 PMC8442456

[7] Wick RR, Judd LM, Gorrie CL, Holt KE. 2017. Completing bacterial genome assemblies with multiplex MinION sequencing. Microb Genom 3:e000132. doi:10.1099/mgen.0.000132. 29177090 PMC5695209

[8] Kolmogorov M, Yuan J, Lin Y, Pevzner PA. 2019. Assembly of long, error-prone reads using repeat graphs. Nat Biotechnol 37:540–546. doi:10.1038/s41587-019-0072-8. 30936562

[9] Wick RR, Holt KE. 2021. Benchmarking of long-read assemblers for proka yote whole genome sequencing. F1000Res 8:2138. doi:10.12688/f1000research.21782.4. PMC6966772 31984131

[10] Vaser R, Šikić M. 2021. Time- and memory-efficient genome assembly with Raven. Nat Comput Sci 1:332–336. doi:10.1038/s43588-021-00073-4. 38217213

[11] Walker BJ, Abeel T, Shea T, Priest M, Abouelliel A, Sakthikumar S, Cuomo CA, Zeng Q, Wortman J, Young SK, Earl AM. 2014. Pilon: an integrated tool for comprehensive microbial variant detection and genome assembly improvement. PLoS One 9:e112963-14. doi:10.1371/journal.pone.0112963. 25409509 PMC4237348

[12] Tanizawa Y, Fujisawa T, Nakamura Y. 2018. DFAST: a flexible prokaryotic genome annotation pipeline for faster genome publication. Bioinformatics 34:1037–1039. doi:10.1093/bioinformatics/btx713. 29106469 PMC5860143

[13] Bortolaia V, Kaas RS, Ruppe E, Roberts MC, Schwarz S, Cattoir V, Philippon A, Allesoe RL, Rebelo AR, Florensa AF, Fagelhauer L, Chakraborty T, Neumann B, Werner G, Bender JK, Stingl K, Nguyen M, Coppens J, Xavier BB, Malhotra-Kumar S, Westh H, Pinholt M, Anjum MF, Duggett NA, Kempf I, Nykäsenoja S, Olkkola S, Wieczorek K, Amaro A, Clemente L, Mossong J, Losch S, Ragimbeau C, Lund O, Aarestrup FM. 2020. ResFinder 4.0 for predictions of phenotypes from genotypes. J Antimicrob Chemother 75:3491–3500. doi:10.1093/jac/dkaa345. 32780112 PMC7662176

